# The genome sequence of the Orange-tipped sea squirt, *Corella eumyota *Traustedt, 1882

**DOI:** 10.12688/wellcomeopenres.21141.1

**Published:** 2024-03-19

**Authors:** John Bishop, Christine Wood, Robert J. Mrowicki, Joanna Harley, Marine Biological Association Genome Acquisition Lab, Darwin Tree of Life Barcoding collective, Wellcome Sanger Institute Tree of Life Management, Samples and Laboratory team, Wellcome Sanger Institute Scientific Operations: Sequencing Operations, Wellcome Sanger Institute Tree of Life Core Informatics team, Tree of Life Core Informatics collective, Darwin Tree of Life Consortium

**Affiliations:** 1 The Marine Biological Association, Plymouth, England, UK

**Keywords:** Corella eumyota, Orange-tipped sea squirt, genome sequence, chromosomal, Phlebobranchia

## Abstract

We present a genome assembly from an individual specimen of *Corella eumyota* (the Orange-tipped sea squirt; Chordata; Ascidiacea; Phlebobranchia; Corellidae). The genome sequence is 129.3 megabases in span. Most of the assembly is scaffolded into 7 chromosomal pseudomolecules. The mitochondrial genome has also been assembled and is 14.53 kilobases in length.

## Species taxonomy

Eukaryota; Opisthokonta; Metazoa; Eumetazoa; Bilateria; Deuterostomia; Chordata; Tunicata; Ascidiacea; Phlebobranchia; Corellidae; *Corella; Corella eumyota* Traustedt, 1882 (NCBI:txid431183).

## Background

**Figure 1.**
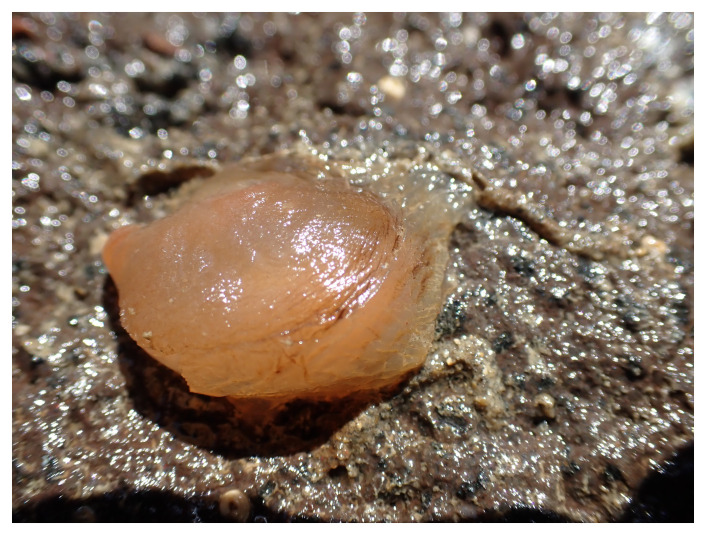
Photograph of *Corella eumyota* by Kelvin Perrie (not the specimen used for genome sequencing).

*Corella eumyota* is a unitary (= ‘solitary’, non-budding) ascidian (sea squirt) ( Figure 1) of the order Phlebobranchia. The flattened, ovate body is typically broadly attached to the substrate by one entire side of the body, and grows to a size of approximately 45 mm. The external covering, or tunic, is smooth, firm, translucent, and frequently remains clear of fouling. The inhalant siphon lies at the extreme anterior end of the body and the exhalant siphon is roughly one third of the way back. The length of the siphons varies extensively according to living conditions: the siphon openings may be barely raised above the general outline of the tunic when the animal has unobstructed access to the water, but longer, slender, poorly contractile siphons may develop in very sheltered and/or crowded conditions. The overall body colour is off-white in specimens growing in dark positions, but with a distinct orange or pinkish-orange tinge when exposed to the light, generally most marked in the siphons (hence the common name Orange-tipped sea squirt). The gut runs around the posterior end of the body in a smooth curve often visible though the tunic.

Unusually for a unitary ascidian, eggs are not spawned for external fertilisation. Instead, they are retained in the atrial chamber, downstream of the branchial basket, where fertilisation and development take place prior to release of the swimming larva through the exhalant siphon ( Lambert *et al.*, 1995). Reproduction seems to involve a mixture of self- and cross-fertilisation ( Dupont *et al.*, 2007).

*C. eumyota* is native to the southern hemisphere, where it occurs on all continents except Antarctica (where it is replaced by one or more close relatives: Alurralde *et al.,* 2013; Alurralde *et al.,* 2018). The broad southern-hemisphere distribution of *C. eumyota* may reflect human-assisted spread from a more restricted natural range. In the northern hemisphere, *C. eumyota* occurs as an apparently rapidly spreading introduced species on the Atlantic coast of north-western Europe, with first recorded occurrences in north-western France in 2002 ( Lambert, 2004), north-western Spain in 2003 ( Varela *et al.*, 2008) and southern England in 2004 ( Arenas *et al.*, 2006).

## Genome sequence report

The genome was sequenced from a specimen of *Corella eumyota* collected from Queen Anne’s Battery Marina Visitors’ Pontoon, Plymouth, Devon, UK (50.36, –4.13). A total of 156-fold coverage in Pacific Biosciences single-molecule HiFi long reads was generated. Primary assembly contigs were scaffolded with chromosome conformation Hi-C data. Manual assembly curation corrected 227 missing joins or mis-joins, reducing the scaffold number by 67.29%, and increasing the scaffold N50 by 98.13%.

**Figure 2.**
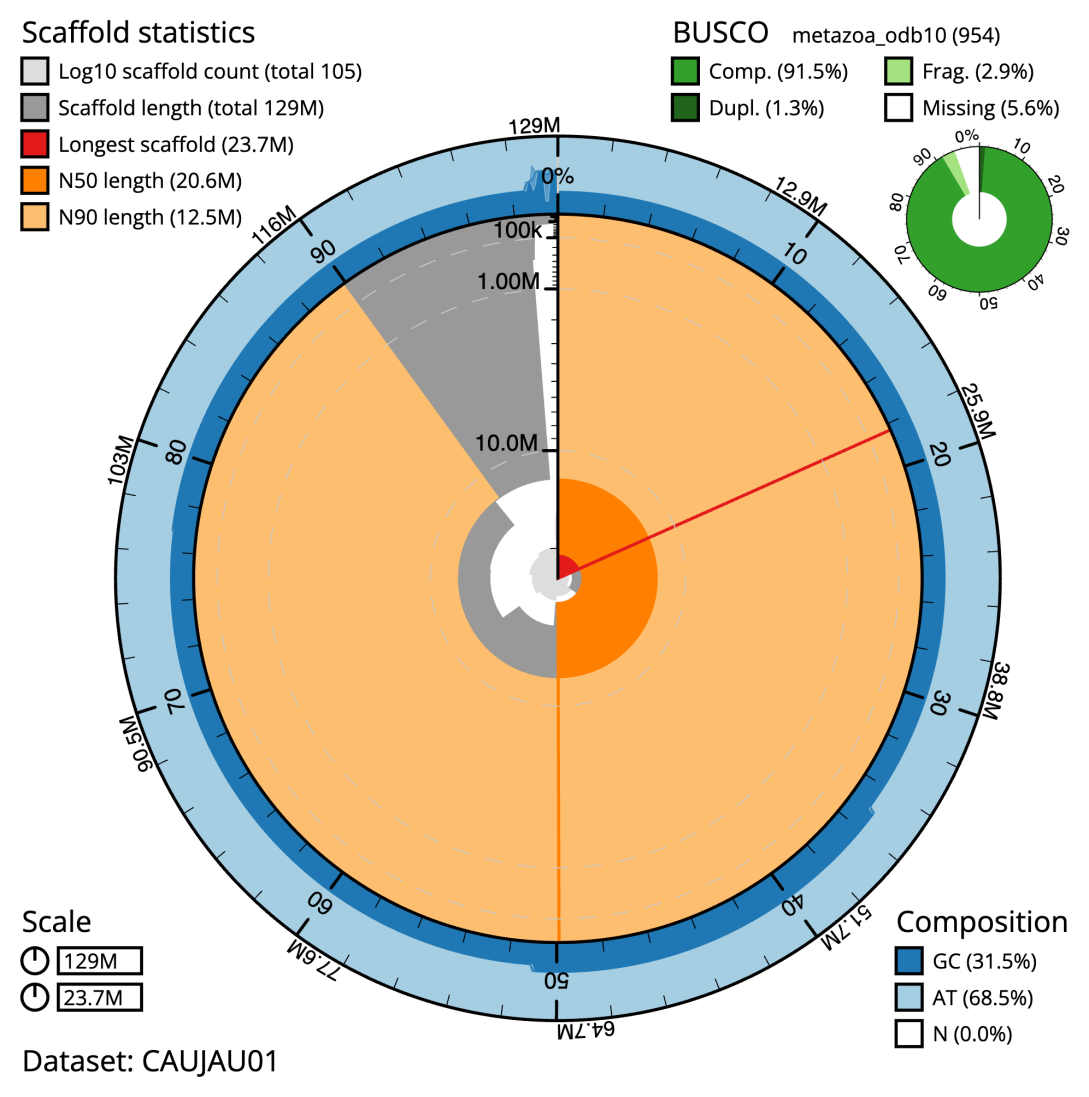
Genome assembly of *Corella eumyota*, kaCorEumy4.1: metrics. The BlobToolKit snail plot shows N50 metrics and BUSCO gene completeness. The main plot is divided into 1,000 size-ordered bins around the circumference with each bin representing 0.1% of the 129,324,498 bp assembly. The distribution of scaffold lengths is shown in dark grey with the plot radius scaled to the longest scaffold present in the assembly (23,716,603 bp, shown in red). Orange and pale-orange arcs show the N50 and N90 scaffold lengths (20,551,778 and 12,493,329 bp), respectively. The pale grey spiral shows the cumulative scaffold count on a log scale with white scale lines showing successive orders of magnitude. The blue and pale-blue area around the outside of the plot shows the distribution of GC, AT and N percentages in the same bins as the inner plot. A summary of complete, fragmented, duplicated and missing BUSCO genes in the metazoa_odb10 set is shown in the top right. An interactive version of this figure is available at https://blobtoolkit.genomehubs.org/view/CAUJAU01/dataset/CAUJAU01/snail.

**Figure 3.**
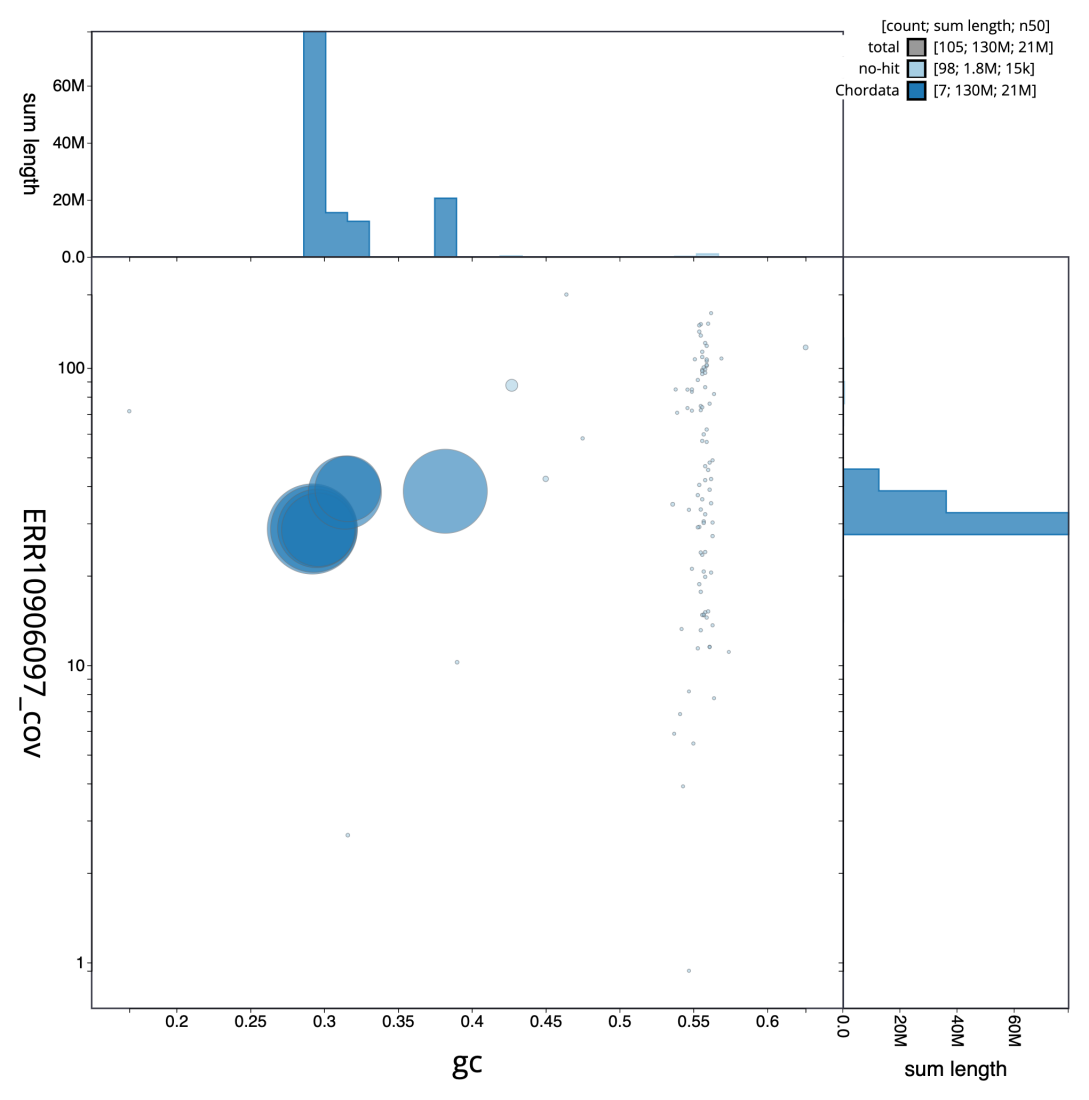
Genome assembly of *Corella eumyota*, kaCorEumy4.1: BlobToolKit GC-coverage plot. Sequences are coloured by phylum. Circles are sized in proportion to sequence length. Histograms show the distribution of sequence length sum along each axis. An interactive version of this figure is available at https://blobtoolkit.genomehubs.org/view/CAUJAU01/dataset/CAUJAU01/blob.

**Figure 4.**
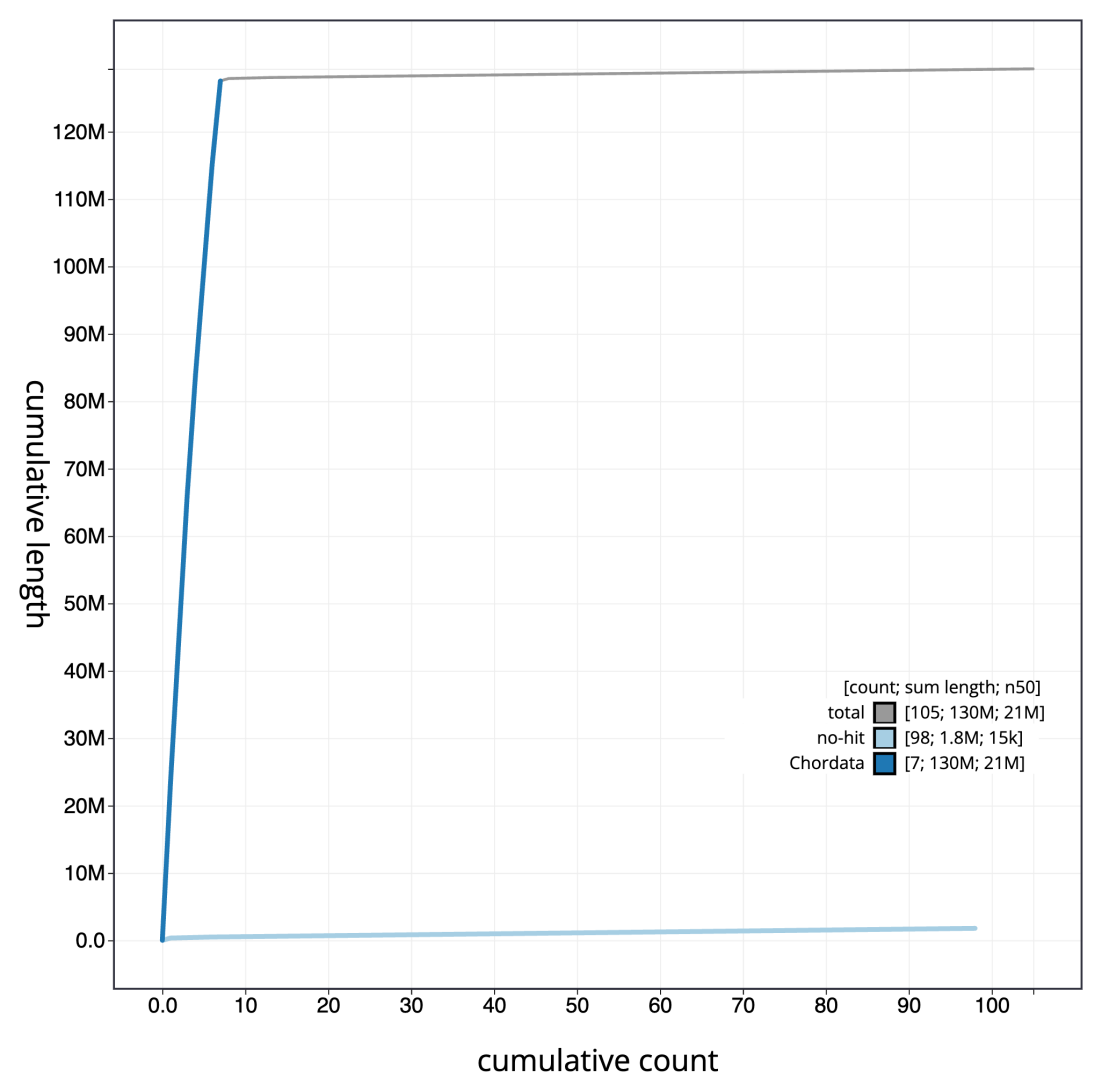
Genome assembly of *Corella eumyota*, kaCorEumy4.1: BlobToolKit cumulative sequence plot. The grey line shows cumulative length for all sequences. Coloured lines show cumulative lengths of sequences assigned to each phylum using the buscogenes taxrule. An interactive version of this figure is available at https://blobtoolkit.genomehubs.org/view/CAUJAU01/dataset/CAUJAU01/cumulative.

**Figure 5.**
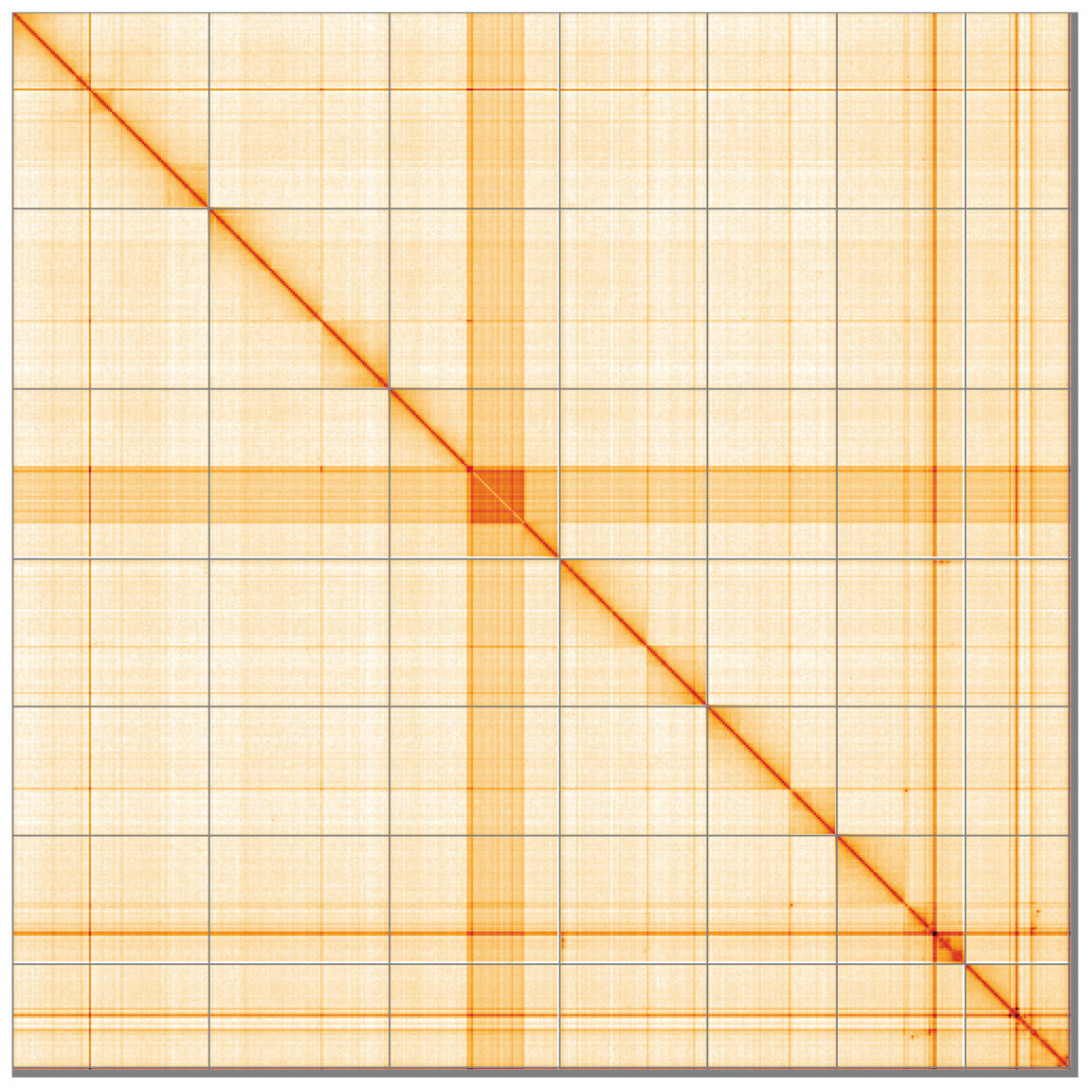
Genome assembly of *Corella eumyota*, kaCorEumy4.1: Hi-C contact map of the kaCorEumy4.1 assembly, visualised using HiGlass. Chromosomes are shown in order of size from left to right and top to bottom. An interactive version of this figure may be viewed at https://genome-note-higlass.tol.sanger.ac.uk/l/?d=Dm63uDhwQlmapSPwvi488A.

The final assembly has a total length of 129.3 Mb in 104 sequence scaffolds with a scaffold N50 of 20.6 Mb ( Table 1). The snail plot in Figure 2 provides a summary of the assembly statistics, while the distribution of assembly scaffolds on GC proportion and coverage is shown in Figure 3. The cumulative assembly plot in Figure 4 shows curves for subsets of scaffolds assigned to different phyla. Most (98.61%) of the assembly sequence was assigned to 7 chromosomal-level scaffolds. Chromosome-scale scaffolds confirmed by the Hi-C data are named in order of size ( Figure 5; Table 2). The order and orientation of contigs along chromosome 3 between 10 Mb and 16.5 Mb are uncertain. While not fully phased, the assembly deposited is of one haplotype. Contigs corresponding to the second haplotype have also been deposited. The mitochondrial genome was also assembled and can be found as a contig within the multifasta file of the genome submission.

**Table 1.** Genome data for *Corella eumyota*, kaCorEumy4.1.

Project accession data		
Assembly identifier	kaCorEumy4.1	
Species	*Corella eumyota*	
Specimen	kaCorEumy4	
NCBI taxonomy ID	431183	
BioProject	PRJEB59956	
BioSample ID	SAMEA7536466	
Isolate information	kaCorEumy4 (DNA, Hi-C and RNA sequencing)	
Assembly metrics *		*Benchmark*
Consensus quality (QV)	65.8	*≥ 50*
*k*-mer completeness	100.0%	*≥ 95%*
BUSCO **	C:91.5%[S:90.3%,D:1.3%], F:2.9%,M:5.6%,n:954	*C ≥ 95%*
Percentage of assembly mapped to chromosomes	98.61%	*≥ 95%*
Sex chromosomes	None	*localised* *homologous pairs*
Organelles	Mitochondrial genome: 14.53 kb	*complete single* *alleles*
Raw data accessions		
PacificBiosciences SEQUEL II	ERR10906097	
Hi-C Illumina	ERR10908634	
PolyA RNA-Seq Illumina	ERR10908635	
Genome assembly		
Assembly accession	GCA_963082875.1	
*Accession of alternate haplotype*	GCA_963082865.1	
Span (Mb)	129.3	
Number of contigs	349	
Contig N50 length (Mb)	5.0	
Number of scaffolds	104	
Scaffold N50 length (Mb)	20.6	
Longest scaffold (Mb)	23.72	

* Assembly metric benchmarks are adapted from column VGP-2020 of “Table 1: Proposed standards and metrics for defining genome assembly quality” from Rhie *et al.* (2021). ** BUSCO scores based on the metazoa_odb10 BUSCO set using version 5.3.2. C = complete [S = single copy, D = duplicated], F = fragmented, M = missing, n = number of orthologues in comparison. A full set of BUSCO scores is available at https://blobtoolkit.genomehubs.org/view/CAUJAU01/dataset/CAUJAU01/busco.

**Table 2.** Chromosomal pseudomolecules in the genome assembly of *Corella eumyota*, kaCorEumy4.

INSDC accession	Chromosome	Length (Mb)	GC%
OY720097.1	1	23.72	29.0
OY720098.1	2	21.79	29.5
OY720099.1	3	20.55	38.0
OY720100.1	4	17.83	29.5
OY720101.1	5	15.63	29.5
OY720102.1	6	15.52	31.5
OY720103.1	7	12.49	31.5
OY720104.1	MT	0.01	17.0

The estimated Quality Value (QV) of the final assembly is 65.8 with *k*-mer completeness of 100.0%, and the assembly has a BUSCO v5.3.2 completeness of 91.5% (single = 90.3%, duplicated = 1.3%), using the metazoa_odb10 reference set ( *n* = 954).

Metadata for specimens, barcode results, spectra estimates, sequencing runs, contaminants and pre-curation assembly statistics are given at https://tolqc.cog.sanger.ac.uk/darwin/chordates/Corella_eumyota/.

## Methods

### Sample acquisition and nucleic acid extraction

A specimen of *Corella eumyota* (specimen ID MBA-200713-003B, ToLID kaCorEumy4) was collected from Queen Anne’s Battery Marina Visitors’ Pontoon, Plymouth, Devon, UK (latitude 50.36, longitude –4.13) on 2020-07-13. The specimen was collected by Christine Wood, John Bishop, Rob Mrowicki and Joanna Harley (Marine Biological Association) and formally identified by Christine Wood and John Bishop. The specimen was collected by hand and placed in a container, and then preserved in liquid nitrogen.

The workflow for high molecular weight (HMW) DNA extraction at the Wellcome Sanger Institute (WSI) includes a sequence of core procedures: sample preparation; sample homogenisation, DNA extraction, fragmentation, and clean-up. In sample preparation, the kaCorEumy4 sample was weighed and dissected on dry ice ( Jay *et al.*, 2023). For sample homogenisation, tissue was cryogenically disrupted using the Covaris cryoPREP ^®^ Automated Dry Pulverizer ( Narváez-Gómez *et al.*, 2023). HMW DNA was extracted using the Manual MagAttract v1 protocol ( Strickland *et al.*, 2023b). DNA was sheared into an average fragment size of 12–20 kb in a Megaruptor 3 system with speed setting 30 ( Todorovic *et al.*, 2023). Sheared DNA was purified by solid-phase reversible immobilisation ( Strickland *et al.*, 2023a): in brief, the method employs a 1.8X ratio of AMPure PB beads to sample to eliminate shorter fragments and concentrate the DNA. The concentration of the sheared and purified DNA was assessed using a Nanodrop spectrophotometer and Qubit Fluorometer and Qubit dsDNA High Sensitivity Assay kit. Fragment size distribution was evaluated by running the sample on the FemtoPulse system.

RNA was extracted from kaCorEumy4 in the Tree of Life Laboratory at the WSI using the RNA Extraction: Automated MagMax™ *mir*Vana protocol ( do Amaral *et al.*, 2023). The RNA concentration was assessed using a Nanodrop spectrophotometer and a Qubit Fluorometer using the Qubit RNA Broad-Range Assay kit. Analysis of the integrity of the RNA was done using the Agilent RNA 6000 Pico Kit and Eukaryotic Total RNA assay.

Protocols developed by the WSI Tree of Life laboratory are publicly available on protocols.io ( Denton *et al.*, 2023).

### Sequencing

Pacific Biosciences HiFi circular consensus DNA sequencing libraries were constructed according to the manufacturers’ instructions. Poly(A) RNA-Seq libraries were constructed using the NEB Ultra II RNA Library Prep kit. DNA and RNA sequencing was performed by the Scientific Operations core at the WSI on Pacific Biosciences SEQUEL II (HiFi) and Illumina HiSeq 4000 (RNA-Seq) instruments. Hi-C data were also generated from tissue of kaCorEumy4 using the Arima2 kit and sequenced on the Illumina NovaSeq 6000 instrument.

### Genome assembly, curation and evaluation

Assembly was carried out with Hifiasm ( Cheng *et al.*, 2021) and haplotypic duplication was identified and removed with purge_dups ( Guan *et al.*, 2020). The assembly was then scaffolded with Hi-C data ( Rao *et al.*, 2014) using YaHS ( Zhou *et al.*, 2023). The assembly was checked for contamination and corrected using the TreeVal pipeline ( Pointon *et al.*, 2023). Manual curation was performed using JBrowse2 ( Diesh *et al.*, 2023), HiGlass ( Kerpedjiev *et al.*, 2018) and PretextView ( Harry, 2022). The mitochondrial genome was assembled using MitoHiFi ( Uliano-Silva *et al.*, 2023), which runs MitoFinder ( Allio *et al.*, 2020) or MITOS ( Bernt *et al.*, 2013) and uses these annotations to select the final mitochondrial contig and to ensure the general quality of the sequence.

A Hi-C map for the final assembly was produced using bwa-mem2 ( Vasimuddin *et al.*, 2019) in the Cooler file format ( Abdennur & Mirny, 2020). To assess the assembly metrics, the *k*-mer completeness and QV consensus quality values were calculated in Merqury ( Rhie *et al.*, 2020). This work was done using Nextflow ( Di Tommaso *et al.*, 2017) DSL2 pipelines “sanger-tol/readmapping” ( Surana *et al.*, 2023a) and “sanger-tol/genomenote” ( Surana *et al.*, 2023b). The genome was analysed within the BlobToolKit environment ( Challis *et al.*, 2020) and BUSCO scores ( Manni *et al.*, 2021; Simão *et al.*, 2015) were calculated.

Table 3 contains a list of relevant software tool versions and sources.

**Table 3.** Software tools: versions and sources.

Software tool	Version	Source
BlobToolKit	4.2.1	https://github.com/blobtoolkit/blobtoolkit
BUSCO	5.3.2	https://gitlab.com/ezlab/busco
Hifiasm	0.16.1-r375	https://github.com/chhylp123/hifiasm
HiGlass	1.11.6	https://github.com/higlass/higlass
Merqury	MerquryFK	https://github.com/thegenemyers/MERQURY.FK
MitoHiFi	2	https://github.com/marcelauliano/MitoHiFi
PretextView	0.2	https://github.com/wtsi-hpag/PretextView
purge_dups	1.2.3	https://github.com/dfguan/purge_dups
sanger-tol/genomenote	v1.0	https://github.com/sanger-tol/genomenote
sanger-tol/readmapping	1.1.0	https://github.com/sanger-tol/readmapping/tree/1.1.0
TreeVal	-	https://github.com/sanger-tol/treeval
YaHS	1.1a.2	https://github.com/c-zhou/yahs

### Wellcome Sanger Institute – Legal and Governance

The materials that have contributed to this genome note have been supplied by a Darwin Tree of Life Partner. The submission of materials by a Darwin Tree of Life Partner is subject to the **‘Darwin Tree of Life Project Sampling Code of Practice’**, which can be found in full on the Darwin Tree of Life website here. By agreeing with and signing up to the Sampling Code of Practice, the Darwin Tree of Life Partner agrees they will meet the legal and ethical requirements and standards set out within this document in respect of all samples acquired for, and supplied to, the Darwin Tree of Life Project. 

Further, the Wellcome Sanger Institute employs a process whereby due diligence is carried out proportionate to the nature of the materials themselves, and the circumstances under which they have been/are to be collected and provided for use. The purpose of this is to address and mitigate any potential legal and/or ethical implications of receipt and use of the materials as part of the research project, and to ensure that in doing so we align with best practice wherever possible. The overarching areas of consideration are:

• Ethical review of provenance and sourcing of the material

• Legality of collection, transfer and use (national and international) 

Each transfer of samples is further undertaken according to a Research Collaboration Agreement or Material Transfer Agreement entered into by the Darwin Tree of Life Partner, Genome Research Limited (operating as the Wellcome Sanger Institute), and in some circumstances other Darwin Tree of Life collaborators.

## Data Availability

European Nucleotide Archive: *Corella eumyota* (orange-tipped sea squirt). Accession number PRJEB59956; https://identifiers.org/ena.embl/PRJEB59956 ( Wellcome Sanger Institute, 2023). The genome sequence is released openly for reuse. The *Corella eumyota* genome sequencing initiative is part of the Darwin Tree of Life (DToL) project. All raw sequence data and the assembly have been deposited in INSDC databases. The genome will be annotated using available RNA-Seq data and presented through the Ensembl pipeline at the European Bioinformatics Institute. Raw data and assembly accession identifiers are reported in Table 1.

## Author information

Members of the Marine Biological Association Genome Acquisition Lab are listed here: https://doi.org/10.5281/zenodo.8382513.

Members of the Darwin Tree of Life Barcoding collective are listed here: https://doi.org/10.5281/zenodo.4893703.

Members of the Wellcome Sanger Institute Tree of Life Management, Samples and Laboratory team are listed here: https://doi.org/10.5281/zenodo.10066175.

Members of Wellcome Sanger Institute Scientific Operations: Sequencing Operations are listed here: https://doi.org/10.5281/zenodo.10043364.

Members of the Wellcome Sanger Institute Tree of Life Core Informatics team are listed here: https://doi.org/10.5281/zenodo.10066637.

Members of the Tree of Life Core Informatics collective are listed here: https://doi.org/10.5281/zenodo.5013541.

Members of the Darwin Tree of Life Consortium are listed here: https://doi.org/10.5281/zenodo.4783558.
