# Supplementary material for: The genome sequence of the Orange-tipped sea squirt, Corella eumyota Traustedt, 1882
Source: Wellcome Open Res. 2024 Oct 11;9:146. Originally published 2024 Mar 19. [Version 2] doi: 10.12688/wellcomeopenres.21141.2 (PMC11494272; doi:10.12688/wellcomeopenres.21141.2)
Supplement: Peer review history

# Reviewer report: Reviewer response for version 2

**DOI:** 10.21956/wellcomeopenres.25602.r104813

**Published:** 2024-10-25

**Authors:** Riccardo Virgili

**Affiliations:** 1 Stazione Zoologica Anton Dohrn, Naples, Italy

The present manuscript "The genome sequence of the Orange-tipped sea squirt, *Corella eumyota* Traustedt, 1882" by Bishop J, Wood C, Mrowicki RJ, Harley J, Marine Biological Association Genome Acquisition Lab, Darwin Tree of Life Barcoding collective, Wellcome Sanger Institute Tree of Life Management, Samples and Laboratory team, Wellcome Sanger Institute Scientific Operations: Sequencing Operations et al. is a detailed report of a new high-quality whole-genome of a phlebobranch ascidian. The manuscript is well-written at this revision stage, not evidencing major flaws. It describes in detail the methodology used for the genome assembly, ensuring a high level of reproducibility.

 I have just a few comments that may improve the manuscript before indexing.

**Is the rationale for creating the dataset(s) clearly described?**

 Partly.

 I particularly appreciated the "background" paragraph, which helps in framing the treated species. I also agree with the author's choice to keep it straight and simple for non-specialists. However, I suggest editing a few points:

 1. As stated by the authors, the length of the siphons is not a stable character and it is not diagnostic within the genus *Corella *(Lambert, 2004). Thus, the relative description should be omitted. Instead, it could be useful to cite the presence of spiralised stigmata in the branchial sac, a character shared only by some Corellidae (Kott 2005; Rocha et al. 2012; Brunetti & Mastrototaro, 2017).

 2. I agree with the authors that the production of high-quality whole genomes (WG) of non-indigenous species is crucial for understanding specific invasion patterns. However, among the applications, it would be also useful to highlight the debate on the systematics of the order Phlebobranchia, recently raised by the WG sequencing of the congeneric *Corella inflata *Huntsman, 1912 (DeBiasse et al. 2020). In this sense, this contribution can be useful for further studies dealing with the paraphyly of phlebobranch ascidians.

 3. The image in Figure 1 does not give proper credit to the species. Being unrelated to the sampled material, clearer CC-BY images of the organisms can be chosen, preferably underwater.

**Are the protocols appropriate and is the work technically sound?**

 Yes

**Are sufficient details of methods and materials provided to allow replication by others?**

 Partly.

 In the methods, the authors state that the specimen was directly placed in liquid nitrogen and then secondarily dissected on dry ice. This method on one side leads to inevitable thawing which is not ideal for the HMW genomic and RNA extractions, and on the other also complicates tissue isolation, which is relevant when working with ascidians to obtaining substantial genomic yields and avoiding contamination.

 In this case, did the authors use the homogenate for the various sequencing steps (including RNAseq), or did they manage to isolate different tissues?

 This information is also lacking in the ERR10908635 details in ENA.

**Are the datasets clearly presented in a useable and accessible format?**

 Yes, with a minor comment.

 I appreciated that the complete mitochondrial genome was assembled and deposited with the chromosome assemblies. There is a general trend in WG reports to upload such resources as unannotated, which partially hinders their utilisation by the community. Mine is just a comment, and I agree with the fact that the aim of this report should be mainly focused on the WG description. However, since, for instance, MitoFinder can provide a quick gene prediction of the assembly in the same pipeline, this resource can be attached as a preliminary annotation, or, in the alternative, the mitogenome can be fully annotated easily.

Are sufficient details of methods and materials provided to allow replication by others?

Partly

Is the rationale for creating the dataset(s) clearly described?

Partly

Are the datasets clearly presented in a useable and accessible format?

Yes

Are the protocols appropriate and is the work technically sound?

Yes

Reviewer Expertise:

ascidian taxonomy, systematics, non-indigenous species

I confirm that I have read this submission and believe that I have an appropriate level of expertise to confirm that it is of an acceptable scientific standard, however I have significant reservations, as outlined above.

## References

[1] Brunetti, R., & Mastrototaro, F. (2017). Ascidiacea of the European Waters. in: Fauna D'Italia. Ediz. Illustrata, vol. LI. Edagricole-New Business Media, Milano, Italy. 446 pp.

[2] : Inferring Tunicate Relationships and the Evolution of the Tunicate Hox Cluster with the Genome of Corella inflata. *Genome Biol Evol* .2020;12(6) : 10.1093/gbe/evaa060 948-964 10.1093/gbe/evaa060 32211845 PMC7337526

[3] Kott, P. (2005). Catalogue of Tunicata in Australian waters. 1. Tunicata - Australia. Australian Biological Resources Study. Canberra, Australian Biological Resources Study.

[4] : The south temperate and Antarctic ascidian Corella eumyota reported in two harbours in north-western France. *Journal of the Marine Biological Association of the UK* .2004;84(1) : 10.1017/S0025315404009105h 239-241 10.1017/S0025315404009105h

[5] : Keys for the identification of families and genera of Atlantic shallow water ascidians. *Biota Neotropica* .2012;12(1) : 10.1590/S1676-06032012000100022 269-303 10.1590/S1676-06032012000100022

# Reviewer report: Reviewer response for version 2

**DOI:** 10.21956/wellcomeopenres.25602.r106373

**Published:** 2024-10-24

**Authors:** Yuki Yoshioka

**Affiliations:** 1 Okinawa Institute of Science and Technology Graduate University Promotion Corp, Onna, Okinawa, Japan

Bishop et al. reported a genome assembly of the Orange-tipped sea squirt at chromosome-scale level. Genomic information is valuable, especially for non-model organisms. The manuscript is well-written. I have only two comments.

 1. Is there any karyotype information? Although I understand that such information is limited in non-model organisms, it strongly supports the result. Please mention it in the manuscript.

 2. Did the author estimate genome size? Please include it in the manuscript.

Are sufficient details of methods and materials provided to allow replication by others?

Yes

Is the rationale for creating the dataset(s) clearly described?

Yes

Are the datasets clearly presented in a useable and accessible format?

Yes

Are the protocols appropriate and is the work technically sound?

Yes

Reviewer Expertise:

Evolutionary biology; Marine genomics;

I confirm that I have read this submission and believe that I have an appropriate level of expertise to confirm that it is of an acceptable scientific standard, however I have significant reservations, as outlined above.

# Reviewer report: Reviewer response for version 2

**DOI:** 10.21956/wellcomeopenres.25602.r104610

**Published:** 2024-10-21

**Authors:** Clara I. Bermúdez-Santana

**Affiliations:** 1 Departamento de Biologia, Universidad Nacional de Colombia,, Bogotá, Bogotá, Colombia

Upon review of the update version of the paper 

 I am now of the opinion that the genome sequence of the orange-tipped sea squirt, *Corella eumyota *Traustedt, 1882, is suitable for indexing. A more detailed description of the assembly, curation and evaluation pipelines, including links to the Tree of Life pipelines used, which were claimed to be included in my first revision, is included in this new version of the data note.

Are sufficient details of methods and materials provided to allow replication by others?

Partly

Is the rationale for creating the dataset(s) clearly described?

Yes

Are the datasets clearly presented in a useable and accessible format?

Partly

Are the protocols appropriate and is the work technically sound?

Yes

Reviewer Expertise:

Comparative genomics

I confirm that I have read this submission and believe that I have an appropriate level of expertise to confirm that it is of an acceptable scientific standard.

# Reviewer report: Reviewer response for version 2

**DOI:** 10.21956/wellcomeopenres.25602.r104819

**Published:** 2024-10-16

**Authors:** Carles Galià Camps

**Affiliations:** 1 Museo Nacional de Ciencias Naturales, Madrid, Community of Madrid, Spain

I enjoyed reading the *Corella eumyota* genome report. It is a pleasure to see the growing number of genomes being sequenced an assembled, specially for such challenging organisms. These efforts are valuable not only from a technical, but also an ecological perspective, given the invasive potential of many ascidian species. I want to congratulate the authors for their initiative and hard work on this project.

 The authors provide a comprehensive description of the species’ morphology to correctly identify it in the field. Following, they underscore the invasive presence of this species the northern hemisphere, and remarks it possible invasive nature in most of the southern hemisphere localities. The methodology followed, is well-documented and robust, resulting into a trusty assembly with exceptional quality. I particularly acknowledge the BUSCO values, which, with scores above 90%, are really good given the complexities of working with tunicates.  

 After carefully reviewing the manuscript, I approve it for indexing. However, I do have a few reservations that, if addresses, would improve the quality of the manuscript, particularly in terms of reproducibility:

  

- **Is the rationale for creating the dataset(s) clearly described?**

Mostly.

 I miss a genetic background. Dupont et al describes *C.* *eumyota* genetic diversity across different regons of the world, being high in New Zeland, low in South Africa, and very low in British waters. I would insert a sentence describing this genetic heterozygosity evidence, fruit of bottlenecks, after the sentence:

 “In the northern hemisphere, *C. eumyota* occurs as an apparently rapidly spreading introduced species on the Atlantic coast of north-western Europe, with first recorded occurrences in north-western France in 2002 ( Lambert, 2004), north-western Spain in 2003 ( Varela *et al.*, 2008) and southern England in 2004 ( Arenas *et al.*, 2006).”

 This would help to understand present-day genetic knowledge, and introduce why this reference genome is necessary.

 Finally, I miss a final sentence in the introduction section specifying how this genome would improve the knowledge about this species at short and long term such as:

  “This assembly will serve as a backbone to understand, through molecular approaches, the colonization routes that *C. eumyota* has used to colonize both hemispheres.”

- **Are the protocols appropriate and is the work technically sound?**

Yes

- **Are sufficient details of methods and materials provided to allow replication by others?**

Mostly, but not fully. Additionally, I found a few inconsistencies. I list below these observations and do some suggestions on how I would improve them:

 - Ascidians inhabit different depth ranges and substrates. You mention that your individuals were collected in a pontoon. However, I would like to know the depth they were inhabiting and if they were attached to a rope (which I guess), the pontoon itself, a ship cask, etc…

 - The manuscript mentions that you sampled one individual, but the metadata refers to four different individuals (from #1 to #4, the latter being used for the genome assembly). I would specify that 4 individuals were collected, 3 of them stored as biobank, and the individual #4, due to its attributes, used for the genome assembly

 - Also, ascidians are known for the difficulties they present when extracting high-quality DNA and RNA. Actually, when I started my PhD I used to work with the syphon, then we moved to the mantle, and we finally conducted the extractions on the gills. Therefore, I would recommend to specify which tissue was used for DNA extraction and Hi-C library preparations. I would also indicate if only one or multiple tissues were used for the RNA extraction. I guess it was more than one tissue or a bulk to capture as much tissue-specific transcripts as possible. If so, specify which tissues were used.

 - I would recommend to add into the M&M section the PacBio HiFi library prep kit name (even if nowadays there is only one kit, it doesn’t mean they can release another one in the future).

 - The manuscript describe that the Hi-C reads were mapped to the primary contigs. However, afterward, in the availability of data, you mention that you only uploaded a haplotype sequence at the chromosome level. I would like to know at which step one of the haplotypes was discarded for chromosome level assembly due to low quality.

 - I like to see all the options used for every software used, as this is the only way to reproduce every step faithfully. I strongly recommend to further describe, for each software, the options used. Otherwise, another possibility is to upload the code in a repository such as GitHub. (optional)

- **Are the datasets clearly presented in a useable and accessible format?**

Yes

- **Other observations (optional)**

I was a little bit shocked since you could not phase both haplotypes. I know that for highly polymorphic genomes this is not accomplishable since information of both haplotypes hamper their obtention during the scaffolding step. However, this is not the case of *C. eumyota*, as Dupont et al. 2007 using microsatellites described an extremely poor heterozygosity in most of the Birttish individuals evaluated in their study.

 I consider it would be interesting to include into the manuscript the heterozygosity of the individual being assembled (0.15%) instead of providing this information into the supplementary. This information, to me, provides a first hint on the difficulty to obtain the two haplotypes. I recommend to include it into the manuscript because I find highly achievable to phase this genome (by yourselves now, in the future, or by another researcher in the future), thus supposing the first phased genome for an ascidian species and expanding its impact.

Are sufficient details of methods and materials provided to allow replication by others?

Partly

Is the rationale for creating the dataset(s) clearly described?

Partly

Are the datasets clearly presented in a useable and accessible format?

Yes

Are the protocols appropriate and is the work technically sound?

Yes

Reviewer Expertise:

Invasive species Genomics, Evolutionary Biologist

I confirm that I have read this submission and believe that I have an appropriate level of expertise to confirm that it is of an acceptable scientific standard, however I have significant reservations, as outlined above.

# Reviewer report: Reviewer response for version 1

**DOI:** 10.21956/wellcomeopenres.23385.r83749

**Published:** 2024-06-18

**Authors:** Federico Brown

**Affiliations:** 1 Departamento de Zoologia, Instituto de Biociências, Universidade de São Paulo, São Paulo, 05508090, Brazil

The manuscript entitled 'The genome sequence of the Orange-tipped sea squirt, *Corella eumyota *Traustedt, 1882' by John Bishop, Christine Wood, Robert J. Mrowicki, Joanna Harley is a flash report of a high quality genome assembly of a phlebobranch solitary ascidian, peculiar for its brooding potential. Highlights of the genome include a relatively small size and low GC%, usual for solitary ascidians, and an assembly that reveals seven putative chromosomes.

 The species description in 'Background' needs to be improved, mainly due to similarities in shape, size and musculature of *C. eumyota* with closely related species *C. brewinae* and *C. antarctica*. As stated a decade ago in a revision of the species (See Monniot, 2013): "As both *Corella* species [ *C. eumyota* and *C. brewinae*] cohabit in the same shore habitat *C.brewinae* while not yet recorded in Europe could also be imported in Europe or in other places in the northern hemisphere like *C. eumyota *was." Thus, despite the absence of reports of these southern species in the Northern hemisphere, a proper description of the diagnostic characters of the sequenced individual in comparison to the above-mentioned species need to be included. Diagnostic characters that have not been described in the manuscript include: length of gonadal ducts and genital papillae, dorsal tubercle, dorsal languets, and number of spiral turns in the stigmata (See Monniot, 2013).

 A brief description of the criteria used for removing contaminants using TreeVal could be specified in the text in besides providing link to the pipeline. Also a brief mention of the nature of contaminants observed would enrich the text.

 I also missed broader comparisons of the *C. eumyota* genome data with other tunicate and animals genomes. In particular, a brief discussion and comparison of the relatively low GC% found across all chromosomal pseudomolecules in *C. eumyota *to other tunicate and animal genomes.

 Last but not least, some of the most interesting questions for data-mining in the *C. eumyota *genome are related to its peculiar brooding behavior, as also recognized by the authors in the 'Background' section. Perhaps the authors could very briefly speculate on what could be some of the expected genomic hallmarks in the *C. eumyota *genome, as a first representative of a solitary ascidian that broods (e.g. oxygen metabolism genes).

Are sufficient details of methods and materials provided to allow replication by others?

Partly

Is the rationale for creating the dataset(s) clearly described?

Partly

Are the datasets clearly presented in a useable and accessible format?

Partly

Are the protocols appropriate and is the work technically sound?

Yes

Reviewer Expertise:

evo-devo, systematics, genomics, developmental biology, evolution, tunicate/ascidian biology

I confirm that I have read this submission and believe that I have an appropriate level of expertise to confirm that it is of an acceptable scientific standard, however I have significant reservations, as outlined above.

## References

[1] : The genus Corella (Ascidiacea, Phlebobranchia, Corellidae) in the Southern Hemisphere with description of a new species. *Zootaxa* .2013;3702: 10.11646/zootaxa.3702.2.3 135-49 10.11646/zootaxa.3702.2.3 26146713

# Reviewer report: Reviewer response for version 1

**DOI:** 10.21956/wellcomeopenres.23385.r83745

**Published:** 2024-06-11

**Authors:** Juan Daniel Montenegro Cabrera

**Affiliations:** 1 University of Vienna, Vienna, Austria

For a genome report it looks ok, there is a rationale for selecting this species to be sequenced and assembled . The methods used for this are correct. However, I think stating that the final construction of pseudomolecules and splitting of mis-join contigs was done "manually" without any description of the parameters or rationale used to split/merge contigs. There must have been some consistency in this part and it should be addressed.

Are sufficient details of methods and materials provided to allow replication by others?

No

Is the rationale for creating the dataset(s) clearly described?

Partly

Are the datasets clearly presented in a useable and accessible format?

Yes

Are the protocols appropriate and is the work technically sound?

Yes

Reviewer Expertise:

Genomics and bioinformatics

I confirm that I have read this submission and believe that I have an appropriate level of expertise to confirm that it is of an acceptable scientific standard, however I have significant reservations, as outlined above.

# Reviewer report: Reviewer response for version 1

**DOI:** 10.21956/wellcomeopenres.23385.r83743

**Published:** 2024-06-04

**Authors:** Tilman Schell

**Affiliations:** 1 Senckenberg Research Institute, Frankfurt am Main, Germany

Review of

 The genome sequence of the Orange-tipped sea squirt, Corella eumyota Traustedt, 1882

 from

 John Bishop, Christine Wood, Robert J. Mrowicki, Joanna Harley, Marine Biological Association Genome Acquisition Lab, Darwin Tree of Life Barcoding collective, Wellcome Sanger Institute Tree of Life Management, Samples and Laboratory team, Wellcome Sanger Institute Scientific Operations: Sequencing Operations, Wellcome Sanger Institute Tree of Life Core Informatics team, Tree of Life Core Informatics collective, Darwin Tree of Life Consortium

 Comments to the authors

 Bishop et al. present the de novo genome assembly of Corella eumyota. PacBio HiFi reads and Arima Hi-C data were utilized to achieve chromosome level scaffolds. The presented analyses show a high quality of the assembly.

 The manuscript is well written and methods are described in a way to ensure reproducibility. Nevertheless, I do have some remarks that need to be clarified before Indexing

 Does the number of chromosome level scaffolds correspond to the karyotype? Is the karyotype known at all? Please add this to the discussion.

 Does the total length of the assembly correspond to genome size estimates? Are there genome size estimates? Please add this to the discussion.

 How were the HiFi reads called? Since you state that a PacBio Sequel II instrument was used, I am wondering if on-board calling was switched on or how HiFi calling was carried out. Please describe in the methods.

 It would be helpful if total Gb and N50 or alike are stated for PacBio HiFi reads used for assembly.

 How were HiFi reads mapped for running purge_dups? Please describe in the methods.

 I would apply contamination screening before Hi-C scaffolding. How does TreeVal identify contamination? If it is based on sequence similarity only, this should be discussed, especially when dealing with non-model organisms, which are underrepresented in public databases.

 How were Hi-C reads mapped for yahs scaffolding? Please describe in the methods.

 The BUSCO scores are not as high as one would expect, given the high quality of the assembly. It would be in your favor to discuss why. I would assume that tunicates are not well represented in the metazoa_odb10 set.

 Were HiFi reads or contigs used as input for MitoHiFi?

 As the other reviewer pointed out already: A comparison to publicly available tunicate genome assemblies would be interesting.

Are sufficient details of methods and materials provided to allow replication by others?

Partly

Is the rationale for creating the dataset(s) clearly described?

Yes

Are the datasets clearly presented in a useable and accessible format?

Yes

Are the protocols appropriate and is the work technically sound?

Yes

Reviewer Expertise:

Genome assembly and annotation

I confirm that I have read this submission and believe that I have an appropriate level of expertise to confirm that it is of an acceptable scientific standard, however I have significant reservations, as outlined above.

# Reviewer report: Reviewer response for version 1

**DOI:** 10.21956/wellcomeopenres.23385.r83748

**Published:** 2024-06-04

**Authors:** Arzu Karahan

**Affiliations:** 1 Institute of Marine Sciences, Middle East Technical University, Erdemli-Mersin, Turkey

It is a well-presented and analysed research.

 I have two concerns;

 First, studying only a single organism is limiting. It would be better to do a genome analysis of at least two individuals, collected from the same or different places.

 Second, working on adult organisms can be problematic. Most ascidians have symbiotic relationships with other species, sometimes including other ascidians. Therefore, it can be challenging to separate the symbiont genes from the target species.

 Additionally, it would be better to include a figure in the text that shows all the pipelines.

Are sufficient details of methods and materials provided to allow replication by others?

Yes

Is the rationale for creating the dataset(s) clearly described?

Yes

Are the datasets clearly presented in a useable and accessible format?

Yes

Are the protocols appropriate and is the work technically sound?

Yes

Reviewer Expertise:

Biodiversity, genomics, tunicates, ageing, eDNA

I confirm that I have read this submission and believe that I have an appropriate level of expertise to state that I do not consider it to be of an acceptable scientific standard, for reasons outlined above.

# Reviewer report: Reviewer response for version 1

**DOI:** 10.21956/wellcomeopenres.23385.r79667

**Published:** 2024-05-15

**Authors:** Clara I. Bermúdez-Santana

**Affiliations:** 1 Departamento de Biologia, Universidad Nacional de Colombia,, Bogotá, Bogotá, Colombia

The genome sequence from a sample of Corella eumyota is reported in this data note. A total of 156x coverage in Pacific Biosciences single molecule HiFi long reads was used to generate an assembly with a total length of 129.3 Mb in 104 sequence scaffolds. The scaffold N50 is 20.6 Mb. Although all raw sequence data and the assembly have been deposited in the INSDC databases, it would be important to have a comparison of the data with other tunicate genomes, for example the size of the genome in comparison to solitary and colonial ascidians. In addition, it needs to be mentioned whether the BUSCO results of C. eumyota are in agreement with previous publications of other comparisons that have been made among other ascidians and metazoans.

Are sufficient details of methods and materials provided to allow replication by others?

Partly

Is the rationale for creating the dataset(s) clearly described?

Yes

Are the datasets clearly presented in a useable and accessible format?

Partly

Are the protocols appropriate and is the work technically sound?

Yes

Reviewer Expertise:

Comparative genomics

I confirm that I have read this submission and believe that I have an appropriate level of expertise to confirm that it is of an acceptable scientific standard, however I have significant reservations, as outlined above.

## References

[1] : Genomic Resources and Annotations for a Colonial Ascidian, the Light-Bulb Sea Squirt Clavelina lepadiformis. *Genome Biol Evol* .2024;16(3) : 10.1093/gbe/evae038 10.1093/gbe/evae038 38441487 PMC10950049

[2] : The Genome of the. *Life (Basel)* .2021;11(12) : 10.3390/life11121377 10.3390/life11121377 34947908 PMC8704543

[3] : The genome sequence of Aplidium turbinatum (Savigny 1816), a colonial sea squirt. *Wellcome Open Res* .2022;7: 10.12688/wellcomeopenres.17785.1 106 10.12688/wellcomeopenres.17785.1 35694195 PMC9171297
